# Social Determinants of Health Screening in Non-Traditional Medical Student Care

**DOI:** 10.51894/001c.164410

**Published:** 2026-08-01

**Authors:** Zachary Kostelec, Mia Shokry, John M. Popovich Jr., Carolina Restini, Lauren Azevedo

**Affiliations:** 1 College of Osteopathic Medicine, Michigan State University

## 52

### Introduction

Social Determinants of Health (SDoH) such as neighborhood, transportation, and food security greatly influence an individual’s overall health. Outside routine one-on-one physician-patient clinical encounters, non-traditional settings (such as health fairs or student clinics) do not have established screening processes to understand community needs.

### Objectives

This project piloted the implementation of SDoH screening using a questionnaire at medical student-run health fairs. We aimed to obtain an accurate understanding of communities’ specific needs, utilize this information to better equip our medical students, and provide a framework for reliable resource connection across different communities.

### Methods

The Community Integrated Medicine organization at Michigan States’ College of Osteopathic Medicine coordinated health fairs which consisted of general health screenings that were anonymous and open to the public. Participants were given the opportunity to complete the American Academy of Family Physicians Social Needs Screening Tool, a 15-item questionnaire to assess common SDoH. The responses for each item were coded as either endorsed (impacted by specific SDoH) or non-endorsed (not impacted by specific SDoH).

### Results

Fifty-eight participants across 4 selected health fairs that focused on 3 separate communities (a non-denominational church dinner n=6, a retired senior citizen volunteer group n=22, and a community center at an Islamic church n=24) completed the screening. In each community, Financial Concerns (33% at The Peoples’ Church, 27% at Foster Grandparents, and 17% at the Islamic Center) and Food Insecurity (17% at The Peoples’ Church, 23% at Foster Grandparents, and 4% at the Islamic Center), were the most frequently endorsed SDoH items. Conversely, SDoH items such as Housing Insecurity were infrequently endorsed (0% - 9%).

### Discussion

The information gained via the anonymous screening allowed medical students to address common SDoH concerns without medical records or repeat patient care. This pilot program improved our process and ability to identify and address SDoH via a tailored resource system. Furthermore, continued tracking of commonly endorsed SDoH items allows health fair resources to be adjusted to best serve the communities’ needs. Next steps include possible research projects in an attempt to identify specific trends across demographic characteristics, including insurance coverage, socioeconomic status, and others.

